# Multimodal Magnetic Nanoparticle–Quantum Dot Composites

**DOI:** 10.3390/nano15241853

**Published:** 2025-12-10

**Authors:** Kareem Ouhalla Knipschild, Vera Kuznetsova, Aoife Kavanagh, Finn Huonder, Caroline O’Sullivan, Amy Clayton, Yaroslav Kryuchkov, Lorenzo Branzi, Yurii K. Gun’ko

**Affiliations:** School of Chemistry, Trinity College Dublin, The University of Dublin, D02 PN40 Dublin, Ireland; ouhallak@tcd.ie (K.O.K.); kavana13@tcd.ie (A.K.); huonderf@tcd.ie (F.H.); osullc45@tcd.ie (C.O.); claytoam@tcd.ie (A.C.); kryuchky@tcd.ie (Y.K.); branzil@tcd.ie (L.B.)

**Keywords:** nanothermometry, ternary quantum dots, CuInS_2_, magnetic nanoparticles, MnFe_2_O_4_, silica encapsulation, multimodal nanocomposites

## Abstract

Multimodal nanocomposites that combine optical and magnetic functionalities are of great interest for applications such as imaging and temperature sensing. Ternary CuInS_2_ (CIS)-based quantum dots (QDs) offer low toxicity, strong near-infrared (NIR) emission, and high photostability, making them promising for optical nanothermometry and imaging. In this study, CIS QDs were synthesized using an aqueous cysteine-mediated approach. Manganese ferrite (MnFe_2_O_4_) nanoparticles were prepared as the magnetic component due to their non-toxicity and superparamagnetic properties. To integrate both functionalities, QDs and magnetic nanoparticles (MNPs) were encapsulated in silica and then combined to form multimodal CIS/MnFe_2_O_4_/SiO_2_ nanocomposites. The structure and morphology of the materials were characterized by TEM and XRD, while their optical properties were examined using UV–Vis, photoluminescence (PL) spectroscopy. This design ensured optical isolation, preventing fluorescence quenching while maintaining colloidal stability. The obtained composites exhibited PL in the NIR region and a thermosensitivity of 2.04%/°C. TEM analysis confirmed uniform silica shell formation and successful integration of both components within the composite. The materials also retained the superparamagnetic behavior of MnFe_2_O_4_, making them suitable for combined optical and magnetic functionalities. These results demonstrate the potential of CIS/MnFe_2_O_4_/SiO_2_ nanocomposites as multifunctional platforms for optical imaging, temperature monitoring, and magnetically modulated effects.

## 1. Introduction

Quantum dots (QDs) are semiconductor nanocrystals (NCs) with size-tunable optical properties arising from quantum confinement effects [1]. Owing to their narrow emission spectra and broad absorption profiles, QDs are widely used in optoelectronic and sensing applications [2]. However, conventional binary QDs such as CdSe or PbS contain toxic heavy metals, which limits their use in practical applications [1,2]. In contrast, ternary I–III–VI QDs, such as CuInS_2_ (CIS) and AgInS_2_ (AIS), offer low toxicity, high photostability, and tunable emission covering the visible to near-infrared (NIR) region, where biological tissues exhibit minimal light absorption, which makes them promising candidates for biological applications [3,4]. Unlike traditional binary QDs, ternary QDs exhibit a different emission mechanism that is primarily governed by donor–acceptor pair recombination and self-trapped excitons; as a result, they display broad photoluminescence (PL) spectra and large Stokes shifts, which significantly reduce reabsorption [5,6,7,8,9,10,11,12]. The incorporation of a ZnS shell around CIS or AIS cores further enhances photoluminescence quantum yield by passivating surface defects and preventing non-radiative recombination [13]. Importantly, the PL intensity of CIS-based QDs is strongly temperature-dependent, as thermal activation enhances non-radiative recombination pathways. This makes CIS QDs a well-established model system for optical thermometry [14,15,16], and their linear PL response in the 25–50 °C range has been reported in previous studies [16].

Magnetic nanoparticles (MNPs) exhibit superparamagnetic behavior at the nanoscale, which allows them to become magnetized under an external magnetic field and demagnetized once the field is removed [17,18]. This reversible magnetization enables external magnetic fields to control particle positioning, alignment, or aggregation in hybrid nanostructures. When exposed to an alternating magnetic field, these particles generate heat through magnetic relaxation mechanisms. This property, combined with the potential for magnetic localization, enables their use in magnetic hyperthermia [19,20]. MnFe_2_O_4_ is widely used in nanoscale magnetic systems due to its superparamagnetic response and high magnetic susceptibility, which originate from its mixed spinel structure [21]. The polyol synthesis method provides colloidally stable, non-aggregated MnFe_2_O_4_ nanoparticles with a reasonably uniform size distribution and optimal particle dimensions [22], making them a suitable magnetic core for uniform multifunctional composites with consistent magnetic and optical performance. Surface functionalization strategies can further improve colloidal stability and compatibility with subsequent processing steps [23]. As a result, MnFe_2_O_4_ serves as an effective magnetic component for field-responsive hybrid materials.

The integration of QDs and MNPs into a single nanocomposite enables the creation of multimodal materials [24] that combine fluorescence, magnetic responsiveness, and temperature sensitivity for multifunctional sensing applications. Such systems can provide localized temperature control and simultaneous optical monitoring and can be used for magnetic nanothermometry, which allows targeted temperature measurement in specific regions where the particles are directed by a magnetic field [25,26,27]. However, direct contact between QDs and MNPs leads to fluorescence quenching due to energy or charge transfer, which can be prevented by spatially separating the components using a dielectric layer [24,26].

In this work, the synthesis of multimodal nanocomposites combining CIS-based QDs with MNPs is described. To prevent fluorescence quenching and ensure colloidal stability, a silica encapsulation strategy was employed, forming core–shell structures that provide optical isolation and high colloidal stability [24,28,29]. The resulting CIS/MnFe_2_O_4_/SiO_2_ composites retained the photoluminescent and thermosensitive properties of the QDs as well as the superparamagnetic characteristics of the magnetic cores, demonstrating their potential for nanothermometry applications.

## 2. Materials and Methods

### 2.1. Materials

(3-Aminopropyl)triethoxysilane (APTES, 98%), ammonia solution (NH_3_, 25%), and indium(III) acetate (In(OAc)_3_, 99.99%) were purchased from Thermo Fisher Scientific, (Dublin, Ireland). (3-Mercaptopropyl)trimethoxysilane (MPTMS, 95%), agarose (molecular biology grade), citric acid (99%), copper(II) acetate (Cu(OAc_2_), 99.99%), ethylene glycol (98%), iron(III) chloride hexahydrate (FeCl_3_·6H_2_O, ≥99%), L-cysteine (L-cys, 97%), sodium sulfide (Na_2_S, 98%), tetraethyl orthosilicate (TEOS, 98%), thioacetamide (TAA, ≥99.0%), trisodium citrate dihydrate (Na_3_C_6_H_9_O_9_·2H_2_O, >99%), and zinc acetate (Zn(OAc)_2_, 99.99%) were purchased from Sigma-Aldrich (Merck Life Science Limited, Arklow, Co., Wicklow, Ireland). Manganese chloride tetrahydrate (MnCl_2_·4H_2_O, 99%) was purchased from Honeywell (Dublin, Ireland). Millipore (MP) water was purified using a Milli-Q water purification system (Merck Life Science Limited, Arklow, Co., Wicklow, Ireland) operating at 18 Ω. All materials were used without further purification.

### 2.2. Quantum Dot Synthesis

#### 2.2.1. CIS QD Synthesis

CIS-based QDs (CuIn_3_S_5_ composition, selected for its optimal balance between thermosensitivity and photoluminescence quantum yield) were synthesized following the procedure reported by Lorenzo Branzi et al. with slight modifications [5,7].

1st step. In 90.0 mL of deionized water, 0.01 M In(AcO)_3_ (18.0 mL, 0.18 mmol) and 0.01 M CuSO_4_ (6.0 mL, 0.06 mmol) were added under constant stirring. The pH of the colorless solution was adjusted to 8.5 with the addition of 1.0 M NaOH (0.6 mL). After vacuuming for 5–10 min, the reaction volume was filled with argon. 0.16 M of optically pure solution of l- or d-cysteine was added (6.0 mL, 0.96 mmol) via syringe and the solution turned colorless. An aqueous solution of 1.0 M Na_2_S was added (0.33 mL, 0.33 mmol, for Cu:In 1:3, resulting in a rapid color change to orange. This solution was kept under constant stirring at room temperature for 1 h. The QDs were precipitated by the addition of 1 M HCl, approximately 1.2–1.3 mL, adjusting the pH to approximately 6, until the particles begin to aggregate. The QDs were collected by centrifugation at 7000-rpm for 1 min and washed three times with deionized water with the addition of 5 uL of 1 M HCl for each washing.

2nd step. The freshly prepared QD pellet was dispersed in 10.0 mL of enantiomerically pure l-cysteine (48.0 mM) solution and a solution of 0.1 M NaOH was used to adjust the pH to 6.3. The QD dispersion was stored in air, for at least 24 h (usually over weekend) to ensure the complete growth of the NCs. Then, the QD dispersions were diluted to a final QD concentration of 1.0 mg mL^−1^ and a ligand concentration of 16.0 mM (diluted 3 times to the final volume 30 mL which can be used for the shell growing).

#### 2.2.2. Core–Shell QD Synthesis

The synthesis was carried out following the method described in the article [13] with some modifications. 1 mg mL^−1^ QD cores (10 mL) were degassed under vacuum for 5 min, then placed under nitrogen and heated to 90/130 °C. A solution of Zn(OAc)_2_ and TAA in distilled water, each at a concentration of 5 mM (0.583 mL) was added to the cores over the course of 90 min at a rate of 0.39 mL h^−1^ via syringe pump. Once this addition was complete, the reaction flask was cooled to room temperature in a water bath. 1 M HCl (33 μL) was added, and once precipitation was visible, the product was separated via centrifugation for 1 min at 9000 RPM and washed 4 times with distilled water. 1 M HCl (2 μL) was added during washing if no precipitation was visible. The product was dispersed in a 10 mL of L-cysteine solution (0.016 M), and the pH was adjusted to 7 using 1 M NaOH (33 μL). The product was stored in a refrigerator at +4 °C.

### 2.3. Magnetitic NP Synthesis

#### 2.3.1. Citrate-Stabilized MnFe_2_O_4_ NP Synthesis

The synthesis followed the procedure reported in the article [22]. FeCl_3_·6H_2_O (2.34 g, 8.7 mmol) and MnCl_2_·4H_2_O (0.86 g, 4.3 mmol) were added to ethylene glycol (50 mL) and mechanically stirred until dissolved. The solution was degassed under vacuum with argon backfill and then kept under argon. Separately, a solution of sodium acetate (3.56 g, 43.4 mmol) in ethylene glycol (50 mL) was prepared and degassed in the same manner. The two degassed solutions were combined under argon and heated to reflux at 198 °C for 20 h while maintaining an inert atmosphere. The solution was cooled to room temperature, magnetically separated, and washed using MP water, methanol, and acetone. The product was stored in ethanol. The product was dried at 80 °C, then an amount (0.50 g, 2.1 mmol) was redissolved in degassed MP water (100 mL) via sonication for 30 min. Trisodium citrate (0.32 g, 1.1 mmol) was added and heated at 80 °C for 2 h. The solution was cooled to room temperature, and 1 g mL^−1^ citric acid solution was added to lower the pH to 7. The product was magnetically separated and stored in degassed MP water (100 mL).

#### 2.3.2. MnFe_2_O_4_/SiO_2_ Core–Shell NP Synthesis

The method described in the article [22] was adapted and modified for this synthesis. Citrate-stabilized MeFe_2_O_4_ solution (4 mL) was diluted with distilled water (8 mL), and HPLC-grade ethanol (40 mL), and 25% ammonia solution (1.25 mL, 18.2 mmol) were added and sonicated for 15 min. The solution was set up for mechanical stirring, and TEOS (250 μL, 1.12 mmol) was added in batches of 25 μL every 30 min. The particles were left to stir for an additional 30 min, following which APTES (1 μL, 4.27 μmol) in ethanol was added and left to stir overnight.

### 2.4. CIS-MnFe_2_O_4_/SiO_2_ Nanocomposite Synthesis

1 mg mL^−1^ CIS 1:3/ZnS QDs (300 μL) were added to ethanol (300 μL) and mixed using magnetic stirring. A 54 mM solution of MPTMS (100 μL, 5.39 μmol) in ethanol was added to the mixture and left to stir for 1 h. A solution of 25% ammonia (2.5 μL) in 1 mL of distilled water (100 μL, 3.66 μmol) was then added and left to stir for an additional hour. The magnetic stirrer was removed, and a solution of SiO_2_-coated MnFe_2_O_4_ from Section 2.3.2 (600 μL) was added. This mixture was shaken in a vortex and then sonicated to break up large aggregates. A 44.4 mM solution of TEOS in ethanol was added (100 μL, 4.48 μmol) and placed into a shaker overnight. The product was magnetically separated and washed twice in ethanol and twice in distilled water, then stored in 1 mL of distilled water.

### 2.5. Equipment

PL was measured using a Horiba Jobin Yvon FluoroMax-4 spectrofluorometer (Horiba, Kyoto, Japan). Thermosensitivity was also measured on this instrument, as well as on a Horiba Jobin Yvon Fluorolog-3 spectrofluorometer. Both fluorometers were equipped with an external temperature-control module. The sample temperature was regulated using an LFI-3751 temperature controller (Wavelength Electronics, Bozeman, MT, USA), which maintained the temperature within ±0.1 °C of the set value. For additional accuracy, the temperature inside the cuvette was independently monitored using an internal thermometer during all measurements. A 1 cm quartz cuvette, an excitation wavelength of 450 nm, and emission and excitation slit widths of 5 nm were employed in both cases. UV–vis data was obtained using an Agilent Technologies Cary-60 UV–Vis spectrophotometer (Cork, Ireland) with a 1 cm quartz cuvette. For thermosensitivity calculation, PL spectra of the CIS QDs were recorded from 10 to 65 °C with 5 °C increments. For each temperature, the PL intensity at the emission peak was extracted, and the degree of PL quenching relative to the initial intensity was calculated. The resulting ΔI/I_0_ values were plotted as a function of temperature. Thermosensitivity was determined as the slope of the linear fit within the 25–50 °C interval, which represents the linear response region. XRD was recorded using a Bruker D2 Phaser benchtop powder diffractometer (Coventry, CV4 8HX, UK) using a Cu Kα radiation source (λ = 1.54184) and a zero-background holder. TEM analysis was carried out using a JEOL JEM-2100 electron microscope (JEOL Ltd., Tokyo, Japan) at 200 kV. The core diameter of CIS QDs was determined from TEM images using ImageJ. Individual particles were measured manually, and at least 50 particles were analyzed for each sample. The mean diameter and standard deviation (mean ± SD) were calculated from the resulting size distributions. SQUID magnetometry was performed using a Quantum Design Ltd. MPMS3 7 Tesla magnetometer (Quantum Design Ltd., Leatherhead, UK) at 300 K, using gelatine capsules to suspend the samples.

## 3. Results and Discussion

### 3.1. QD Synthesis and Structural Characterization

CIS QDs were obtained using the synthesis reported previously by Branzi et al. [7], an aqueous synthesis which uses L-cysteine, an amino acid which produces a colloidal solution in aqueous media, as a capping agent, where it binds to the QD surface and passivates the exposed surface sites. It also allows for further functionalization via its negative surface charge. This was followed by a zinc sulfide shelling step. The synthesis was carried out under inert conditions, due to the vulnerability of copper (I) to oxidation.

**Figure 1:**
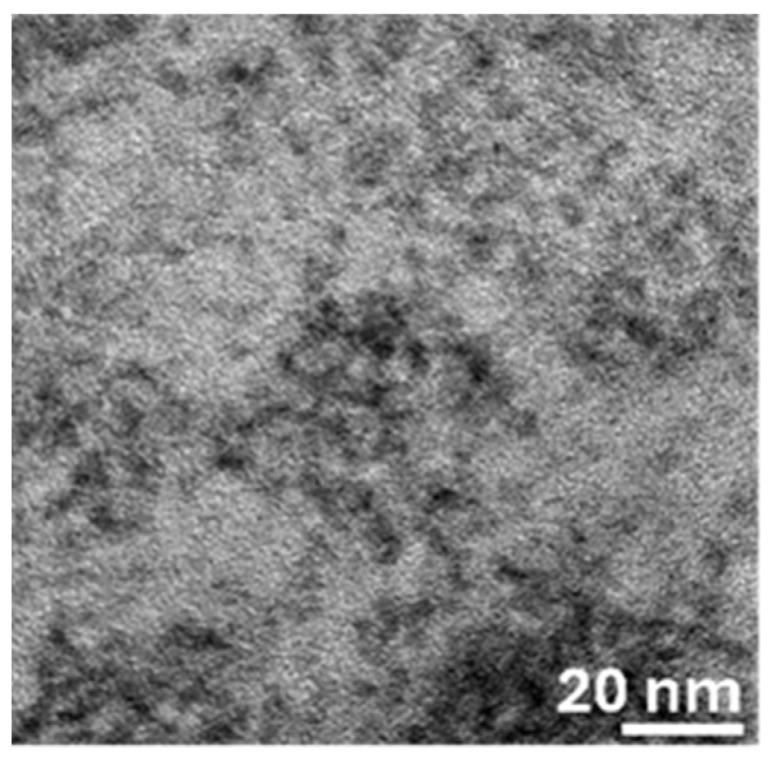
TEM images of CIS QDs.

TEM analysis was carried out on the shelled CIS QDs (Figure 1). Despite being somewhat agglomerated, which is typical in aqueous QDs due to strong capillary forces while drying [30], several individual QDs could be identified. A size distribution of 3.0 ± 0.4 nm was obtained, which is typical for aqueous CIS QDs [13]. The size distribution histogram is provided in the Supplementary Material (SI), Figure S1.

**Figure 2:**
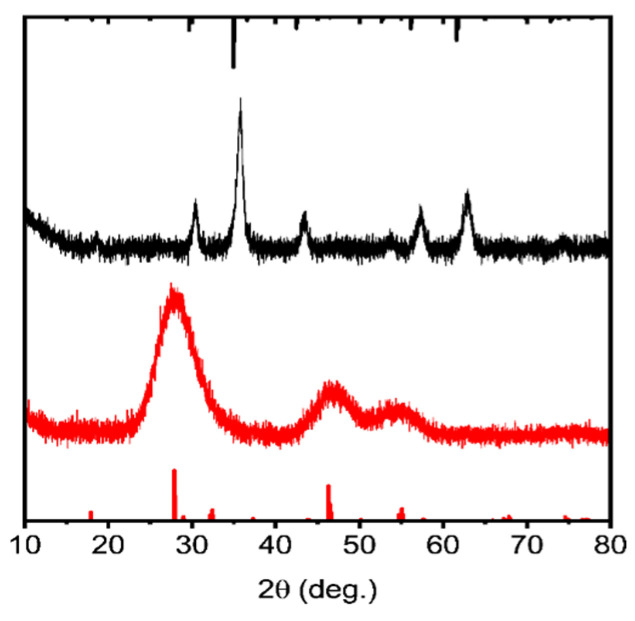
XRD pattern of MnFe_2_O_4_ MNP (black) and CIS13 QDs (red). Reference patterns: MnFe_2_O_4_ Fd-3m COD1010371 (black) CuInS2 I-42d ICSD66865 (red).

XRD patterns of CIS QDs are presented in Figure 2. Due to the very small size of the QDs, broad peaks are observed in their diffraction patterns. XRD analysis confirms that CIS QDs adopt the tetragonal chalcopyrite structure. The large peak at a 2θ of approx. 28° relates to the (4 1 1) plane, and d-spacing was 3.19 Å.

### 3.2. Optical Characterization of QDs

#### 3.2.1. UV–Vis Spectroscopy

**Figure 3:**
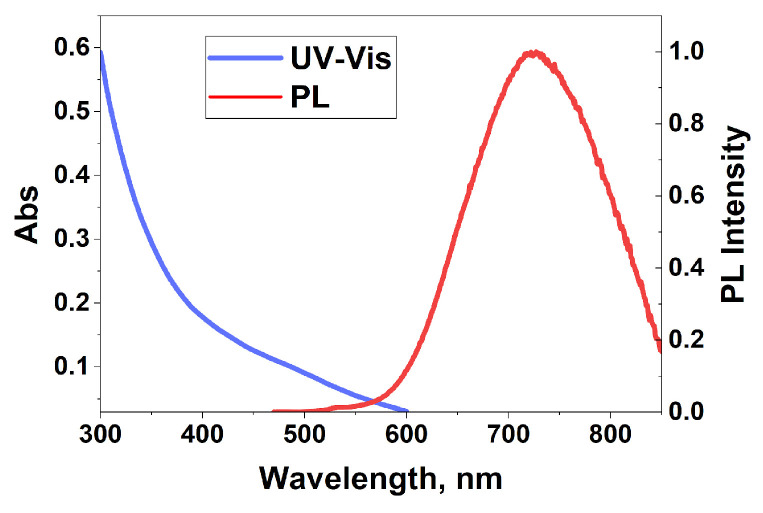
UV–vis and PL emission spectra of CIS QDs.

The UV–vis spectrum of CIS QDs were measured at an approximate concentration of 33 μg/mL. The spectrum exhibited a broad absorption across the UV–vis. (Figure 3). A broad peak at ~500 nm can be seen. This is an excitonic transition, which broadens in CIS QDs due to defect states within the band [31].

#### 3.2.2. Photoluminescence Spectroscopy

Figure 3 shows the PL spectra of the QDs. The PL peak position was 708 nm, with a full width at half maximum of 162 nm. Importantly, the emission occurs in the NIR region, which is favorable for biomedical applications due to the low absorbance of biological tissue in this range. Additionally, the samples exhibit a large Stokes shift, minimizing self-absorption and further enhancing their suitability for bioimaging applications [12].

### 3.3. Thermosensitivity Measurements

**Figure 4:**
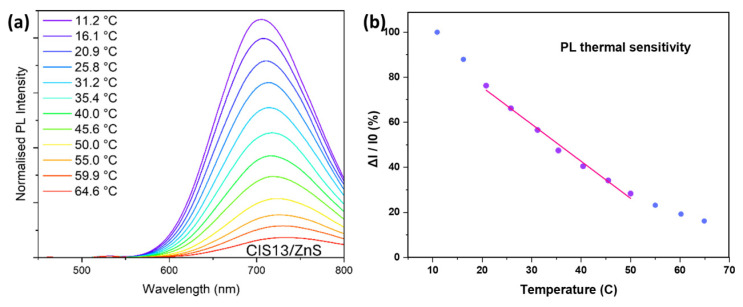
Thermosensitivity of CIS QDs: (**a**) PL spectra of CIS QDs in the temperature range from 10 to 65 °C with a 5 °C step. (**b**) Temperature dependence of the PL quenching degree (ΔI/I_0_), showing a linear decrease in PL intensity with increasing temperature in the 25–50 °C region. Linear approximation is shown in red.

Thermosensitivity is a key property in determining the suitability of QDs for nanothermometry applications. To assess this property, we recorded the PL spectra of the CIS QDs in the temperature range from 10 to 65 °C with a 5 °C step (Figure 4a) and constructed the temperature dependence of the PL quenching degree (ΔI/I_0_) (Figure 4b). On the 25–50 °C interval, the PL intensity of CIS-based QDs decreases linearly with temperature, making this range suitable for accurate measurements, which is consistent with the data reported by Zhang et al. [16]. The details of the linear approximation are provided in the SI.

The thermosensitivity value was 2.09 ± 0.12 ΔI_%_/°C. The optical properties of CIS have been linked to both defect states causing donor–acceptor recombination within the bandgap, as well as to electron-phonon coupling.

### 3.4. Multimodal Nanocomposite

**Figure 5:**
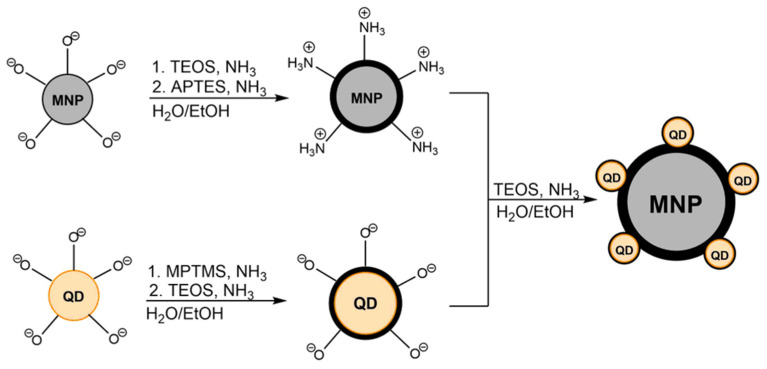
Schematic representation of silica encapsulation approach to composite preparation.

The multimodal composites were assembled by first encapsulating the MNPs and QDs separately in silica and then combining them. This strategy provided sufficient spatial separation between the components to avoid fluorescence quenching and improved the overall colloidal stability of the system (Figure 5).

**Figure 6:**
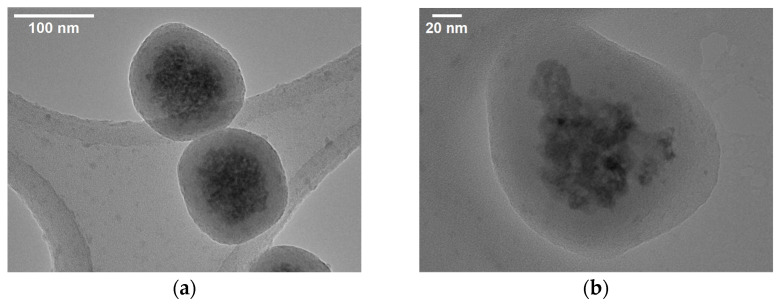
(**a,b**) TEM images of CIS 1:3/MnFe_2_O_4_/SiO_2_ composite.

Manganese ferrite nanoparticles were synthesized by the polyol method and stabilized with citrate ions using the procedure described in article [22]. This approach yielded MnFe_2_O_4_ nanoparticles with an average size of 74.1 ± 14.5 nm (Figure 6 and SI). XRD patterns of the MnFe_2_O_4_ nanoparticles are shown in Figure 2. The diffraction peaks correspond to the characteristic reflections of the spinel ferrite phase, including the (220), (311), (400), (511), and (440) planes. The most intense peak at approximately 2θ ≈ 35° is assigned to the (311) reflection, which is typical for MnFe_2_O_4_. The particles were subsequently coated with a silica shell. The citrate ions on the surface provided colloidal stability, enabling the formation of non-aggregated individual magnetic nanoparticles encapsulated within a silica (Figure 6). For the silica coating, TEOS was added to citrate-stabilized manganese ferrite nanoparticles. Ammonia solution was added to catalyze the hydrolysis of the methoxy groups and form a strong Si-O bond with surface oxygens on the magnetite. APTES then was added, once again in the presence of ammonia, forming an additional shell to provide a positive surface charge to the particles. A second silica shell was then constructed around the QDs. The QDs were combined with MPTMS, which can adsorb onto the surface of the QDs via its thiol group, which was shown not to lead to quenching. This led to the formation of a silica shell around the QDs. TEOS was added to thicken the silica shell. As silica has a negative charge due to hydroxy groups, it allowed for composite formation via electrostatic interactions.

The silica shelled MNPs and QDs were then combined, and the product was magnetically separated and washed several times in ethanol and distilled water. Noticeably, it retained its luminescence even after washing. TEM analysis was carried out on the composites (Figure 6).

Several colloidal particles can be seen with a size distribution of 125.6 ± 21.9 nm. In each case, a contrast can be seen between the outer silica shell and the interior. While the QDs are not immediately apparent, a feature can be observed in many of the particles, such as in Figure 6b, where a part of the otherwise spherical particle protrudes outwards. This is possibly an effect of the silica-shelled QD attaching to the larger silica-shelled MNP.

**Figure 7:**
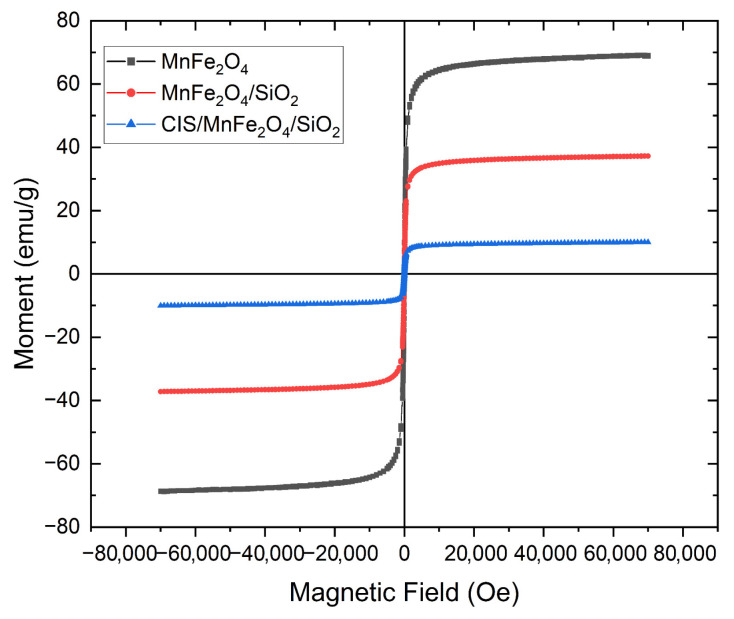
SQUID-VSM magnetization curves of MnFe_2_O_4_, MnFe_2_O_4_/SiO_2_ and CIS/MnFe_2_O_4_/SiO_2_ at 300 K.

The magnetic properties of the CIS/MnFe_2_O_4_/SiO_2_ composite was investigated by vibrating sample magnetometry (VSM) using a superconducting quantum interference device (SQUID) at 300 K. The citrate-stabilized MnFe_2_O_4_ MNPs displayed superparamagnetic behavior, as evidenced by the absence of coercivity and remanence in their magnetization curve (Figure 7), and exhibited a saturation magnetization of 69 emu/g. Upon functionalization of the MNPs with a SiO_2_ outer shell, the profile of their magnetization curve remained unchanged, however there was an expected decrease in the saturation magnetization to 37 emu/g, attributed to the presence of non-magnetic SiO_2_. Subsequent functionalization of the MnFe_2_O_4_/SiO_2_ MNPs with CIS/SiO_2_ resulted in a further decrease in saturation magnetization to 10 emu/g, yet the material remained superparamagnetic in nature.

**Figure 8:**
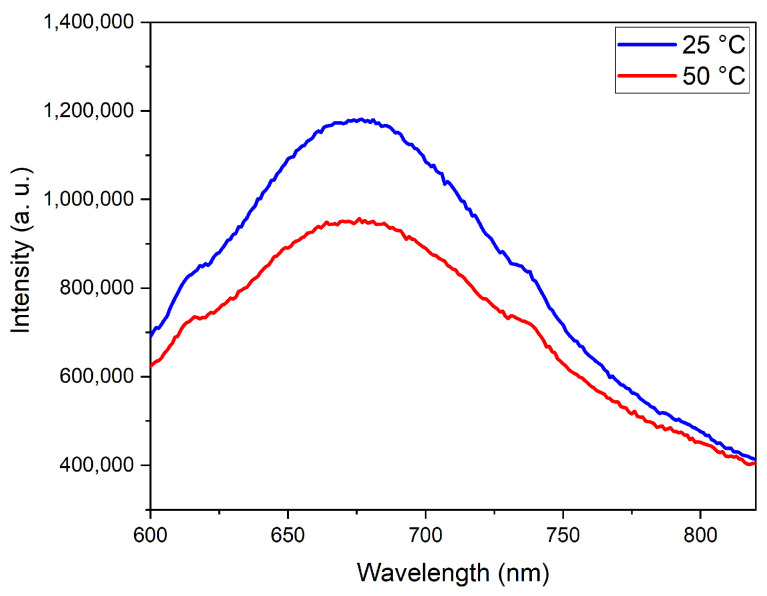
Demonstration of thermosensitivity of CIS 1:3/MnFe_2_O_4_/SiO_2_ composite using PL spectroscopy.

To definitively confirm the presence of the QDs in the composite, PL and thermosensitivity measurements were carried out (Figure 8).

While significant scattering can be seen due to the relatively large particle size, a high PL intensity is exhibited by the composite. A thermosensitivity of 2.04% per degree was calculated, which is close to the sensitivity of the CIS QDs, which was determined to be 2.09% per degree, showing that the QDs had mostly retained their sensitivity. Thus, this was a very promising result with a potentially suitable multimodal magnetic–fluorescent nanocomposite.

## 4. Conclusions

In this work, multimodal CIS/MnFe_2_O_4_/SiO_2_ nanocomposites were successfully synthesized by integrating CIS-based QDs with manganese ferrite nanoparticles through a silica encapsulation strategy. The silica shell provided effective spatial separation between the optical and magnetic components, preventing fluorescence quenching and ensuring good colloidal stability. The resulting composites exhibited strong NIR PL, high thermal sensitivity comparable to that of pristine CIS QDs, and preserved superparamagnetic behavior. These findings confirm that silica-encapsulated CIS/MnFe_2_O_4_ nanostructures combine optical and magnetic functionalities within a single platform, offering significant potential for applications in magnetic hyperthermia, temperature sensing, and theranostic systems.

## Data Availability

Data are available from the authors on demand.

## Supplementary Materials

The following supporting information can be downloaded at https://www.mdpi.com/article/10.3390/nano15241853/s1, Figure S1. TEM images of CIS QDs (a) and size distribution diagram (b). The average size was 3.0 ± 0.4 nm. Figure S2. TEM images of MPTMS/TEOS shelled CIS QDs (a) and size distribution diagram (b). An average size was 6.7 ± 1.1 nm. Figure S3. TEM images of CIS 1:3/MnFe_2_O_4_/SiO_2_ composite (a) and size distribution diagrams of (b) MnFe_2_O_4_ nanoparticles with an average size of 74.1 ± 14.5 nm and (c) CIS 1:3/MnFe_2_O_4_/SiO_2_ composite with an average size of 125.6 ± 21.9 nm. Figure S4. Linear approximation of the PL quenching degree (ΔI/I_0_) of CIS QDs as a function of temperature in the 25–50 °C interval (R^2^ = 0.989).

## Abbreviations

The following abbreviations are used in this manuscript:

CIS	Copper Indium Sulfide
QDs	Quantum Dots
NCs	Nanocrystals
MNPs	Magnetic Nanoparticles
UV–Vis	Ultraviolet–Visible spectroscopy
PL	Photoluminescence
TEM	Transition Electron Microscopy
XRD	X-ray Diffraction
VSM	Vibrating Sample Magnetometry
SQUID	Superconducting Quantum Interference Device
