# Supplementary material for: Multimodal Magnetic Nanoparticle–Quantum Dot Composites
Source: Nanomaterials (Basel). 2025 Dec 10;15(24):1853. doi: 10.3390/nano15241853 (PMC12735848; doi:10.3390/nano15241853)
Supplement: Supplementary file 1 [file nanomaterials-15-01853-s001.zip › nanomaterials-3992260-supplementary.pdf]

Article

Multimodal Magnetic Nanoparticle–Quantum Dot Composites

Kareem Ouhalla Knipschild, Vera Kuznetsova *, Aoife Kavanagh, Finn Huonder, Caroline O'Sullivan, Amy Clayton, Yaroslav Kryuchkov, Lorenzo Branzi and Yurii K. Gun'ko *

School of Chemistry, Trinity College Dublin, The University of Dublin, D02 PN40 Dublin, Ireland; ouhallak@tcd.ie (K.O.K.); kavana13@tcd.ie (A.K.); huonderf@tcd.ie (F.H.); osullc45@tcd.ie (C.O.); claytoam@tcd.ie (A.C.); kryuchky@tcd.ie (Y.K.); branzil@tcd.ie (L.B.)

* Correspondence: kuznetsv@tcd.ie (V.K.); igounko@tcd.ie (Y.K.G.)

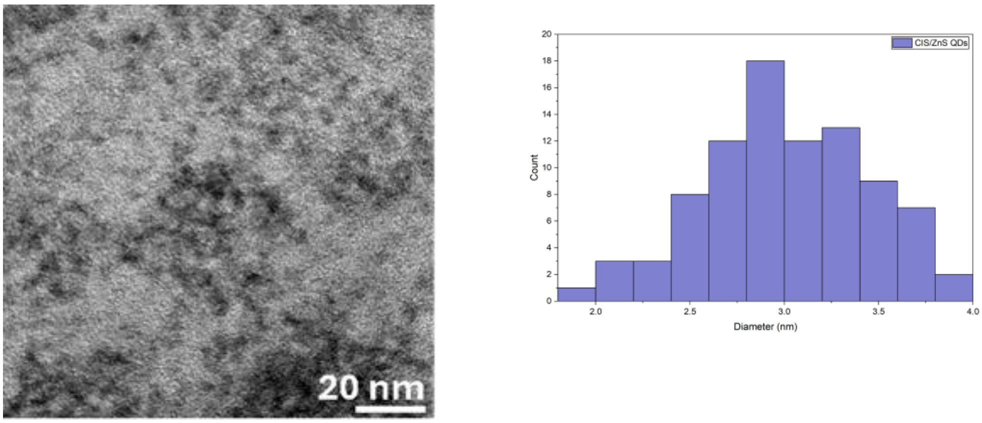

(**a**) (**b**)

Figure S1. TEM images of CIS QDs (**a**) and size distribution diagram (**b**). The average size was 3.0 ± 0.4 nm.

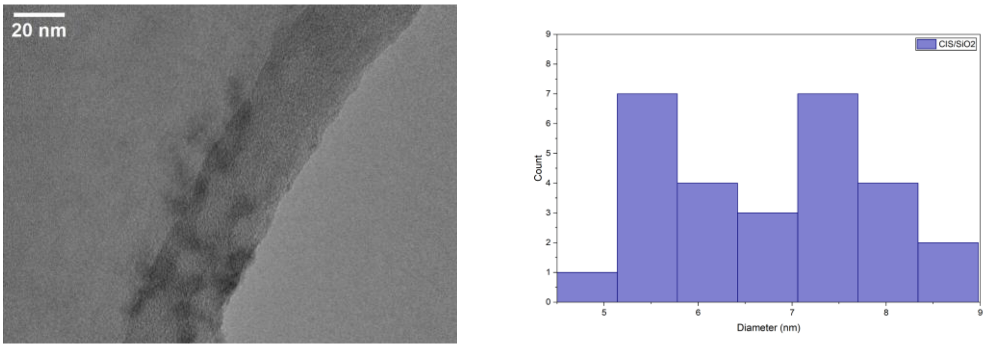

(a) **(b)**

Figure S2. TEM images of MPTMS/TEOS shelled CIS QDs (**a**) and size distribution diagram (**b**). An average size was 6.7 ± 1.1 nm.

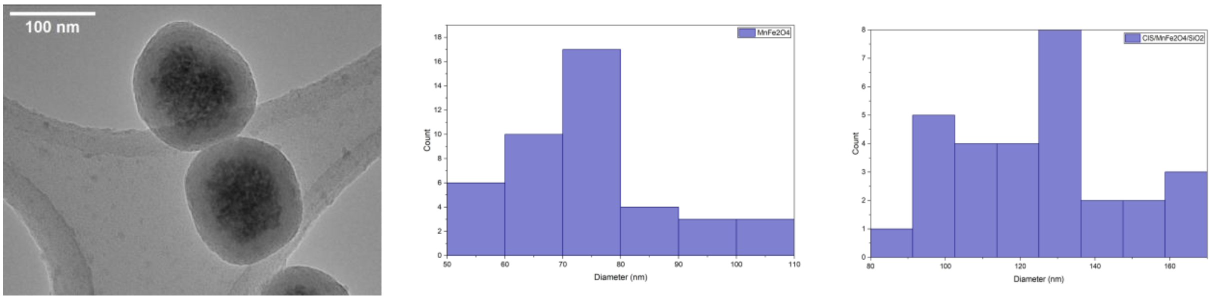

(a) **(b)** **(c)**

Figure S3. TEM images of CIS 1:3/MnFe2O4/SiO2 composite (**a**) and size distribution diagrams of (**b**) MnFe2O4 nanoparticles with an average size of 74.1 ± 14.5 nm and (**c**) CIS 1:3/MnFe2O4/SiO2 composite with an average size of 125.6 ± 21.9 nm.

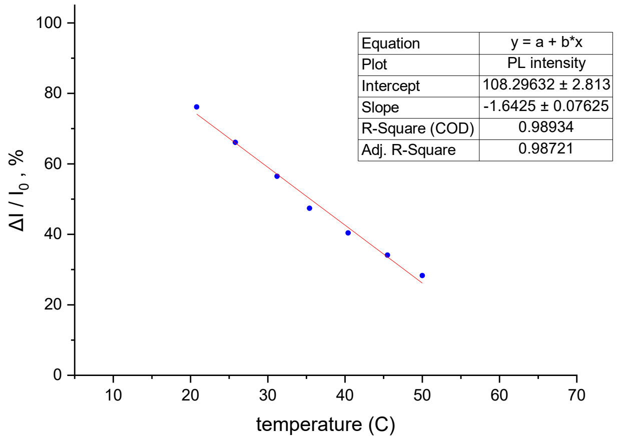

Figure S4. Linear approximation of the PL quenching degree ($\Delta I/I_0$) of CIS QDs as a function of temperature in the 25–50 °C interval (R^2 = 0.989).
